# Engineered *Escherichia coli* cell factory for anthranilate over-production

**DOI:** 10.3389/fmicb.2023.1081221

**Published:** 2023-03-15

**Authors:** Hye-Jin Kim, Seung-Yeul Seo, Heung-Soon Park, Ji-Young Ko, Si-Sun Choi, Sang Joung Lee, Eung-Soo Kim

**Affiliations:** ^1^Department of Biological Sciences and Bioengineering, Inha University, Incheon, Republic of Korea; ^2^STR Biotech Co., Ltd., Gangwon-do, Republic of Korea

**Keywords:** Anthranilate, cell factory design, *Escherichia coli*, metabolic engineering, genome editing

## Abstract

Anthranilate is a key platform chemical in high demand for synthesizing food ingredients, dyes, perfumes, crop protection compounds, pharmaceuticals, and plastics. Microbial-based anthranilate production strategies have been developed to overcome the unstable and expensive supply of anthranilate *via* chemical synthesis from non-renewable resources. Despite the reports of anthranilate biosynthesis in several engineered cells, the anthranilate production yield is still unsatisfactory. This study designed an *Escherichia coli* cell factory and optimized the fed-batch culture process to achieve a high titer of anthranilate production. Using the previously constructed shikimate-overproducing *E. coli* strain, two genes (*aroK* and *aroL*) were complemented, and the *trpD* responsible for transferring the phosphoribosyl group to anthranilate was disrupted to facilitate anthranilate accumulation. The genes with negative effects on anthranilate biosynthesis, including *pheA*, *tyrA*, *pabA*, *ubiC*, *entC*, and *trpR*, were disrupted. In contrast, several shikimate biosynthetic pathway genes, including *aroE* and *tktA*, were overexpressed to maximize glucose uptake and the intermediate flux. The rationally designed anthranilate-overproducing *E. coli* strain grown in an optimized medium produced approximately 4 g/L of anthranilate in 7-L fed-batch fermentation. Overall, rational cell factory design and culture process optimization for microbial-based anthranilate production will play a key role in complementing traditional chemical-based anthranilate production processes.

## Introduction

Anthranilate is a key metabolite involved in the aromatic amino acid biosynthetic pathway and a vital metabolite used as a precursor for the synthesis of many valuable aromatic compounds in bacteria, fungi, and plants (Jiang and Zhang, 2016; Averesch and Krömer, 2018; Santos-Sánchez et al., 2019). These aromatic amino acids, such as L-phenylalanine (L-Phe), L-tyrosine (L-Tyr), and L-tryptophan (L-Trp), which are typically synthesized *via* the shikimate pathway, are used as precursors for the production of various secondary metabolites essential for plant growth, and also critical building blocks for protein biosynthesis in microorganisms (Weaver and Herrmann, 1997; Tzin and Galili, 2010; Jiang and Zhang, 2016; Santos-Sánchez et al., 2019).

**Figure 1:**
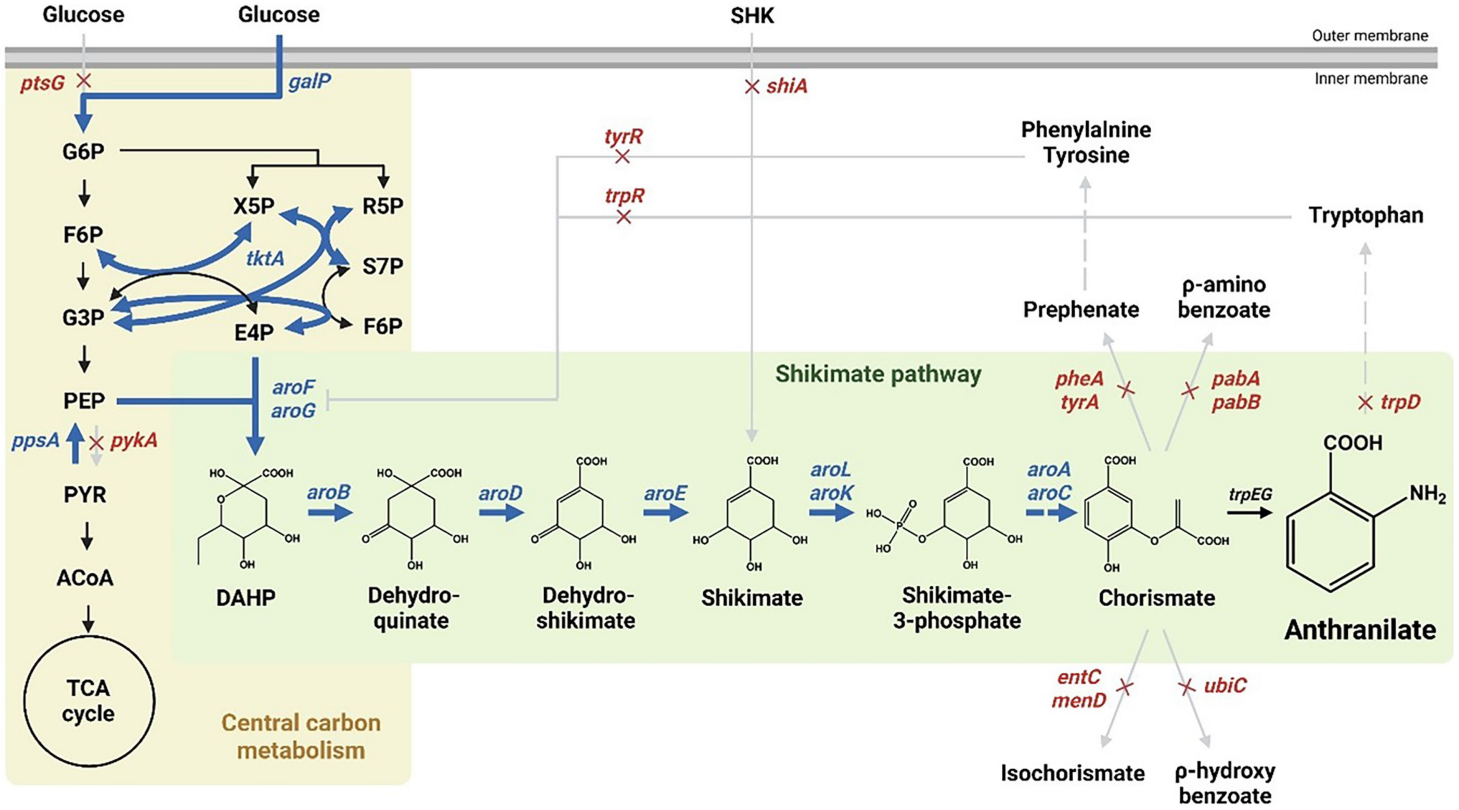
Scheme of the pathway engineering for anthranilate production in *Escherichia coli*. Red crosses indicate disrupted genes and bold blue arrows indicate enhanced steps through target gene overexpression. Dashed arrows represent two or more steps. G6P; glucose-6-phosphate, F6P; fructose-6-phosphate, G3P; glyceraldehyde-3-phosphate, PEP; phosphoenolpyruvate, PYR; pyruvate, ACoA; acetyl-CoA, X5P; xylulose-5-phosphate, R5P; ribose-5-phosphate, S7P; sedoheptulose-7-phosphate, E4P; erythrose-4-phosphate, F6P; fructose-6-phosphate, DAHP; 3-deoxy-D-arabisoheptulosanate-7-phosphate.

The shikimate pathway generates chorismate through sequential enzymatic reactions, starting with a condensation reaction of phosphoenolpyruvate (PEP) produced *via* the glycolic pathway and erythrose-4-phosphate (E4P) produced *via* the pentose phosphate pathway (Jiang and Zhang, 2016; Averesch and Krömer, 2018; Santos-Sánchez et al., 2019). In *E. coli*, chorismate is converted to prephenate, anthranilate, aminodeoxychorismate, isochorismate, and x-hydroxybenzoate in five enzymatic reactions used in the production of various aromatics, including aromatic amino acids (Figure 1, Dosselaere and Vanderleyden, 2001; Gosset, 2009; Koma et al., 2012; Noda et al., 2016).

Anthranilate is an aromatic acid used as a platform chemical for the production of food ingredients, dyes, perfumes, crop protection compounds, pharmaceutical compounds, and plastics (Askham, 1992; Yadav and Krishnan, 1998; Wiklund and Bergman, 2006; Balderas-Hernández et al., 2009; Bahia et al., 2011; Shafiq et al., 2011; Haynes et al., 2012; Chambers et al., 2013; Gao et al., 2013; Loque and Weniger, 2013; Sun et al., 2013; Walsh et al., 2013; Kuepper et al., 2015; Lee et al., 2019; Luo et al., 2019). In addition, anthranilate inhibits biofilm formation in a wide range of bacteria, which is expected to expand the industrial application of anthranilate (Li et al., 2017). Since shikimate was recently reported to stimulate hair growth through cell proliferation of human dermal papilla cells and outer root sheath cells (Choi M. et al., 2019; Choi N. et al., 2019), potential value of shikimate pathway metabolites including anthranilate also continues to expand.

Currently, anthranilate production is based on chemical synthesis using petroleum-derived precursors, such as benzene, which is an energy-intensive process limited by toxic byproduct production (Balderas-Hernández et al., 2009; Kuepper et al., 2015). Therefore, attempts are being made to produce anthranilate from renewable resources in an environmentally friendly manner, and microbial-based anthranilate production is being suggested as an alternative. According to previous studies, up to 14 g/L of anthranilate production was reported through the fed-batch fermentation of recombinant *E. coli* strain W3110 *trpD*9923/pJLBaroG^fbr^tktA, and 1.5 g/L of anthranilate production was achieved using *Pseudomonas putida* KT2440 strain (*P. putida* △*trpDC*/pSEVA234_*aroG^D146N^*_*trpE^S40F^G*), respectively (Balderas-Hernández et al., 2009; Kuepper et al., 2015; Fernández-Cabezón et al., 2022). A genome-engineered *Corynebacterium glutamicum* YTM5 strain harboring pEKGH produced 26.40 g/L of anthranilate in glucose minimal medium (Luo et al., 2019). An engineered yeast strain was also reported to produce 0.56 g/L of anthranilate (Kuivanen et al., 2021).

Previous studies published the world’s highest shikimate high-production *E. coli* strain. The current study attempted to develop an anthranilate high-production strain by optimizing the shikimate-to-anthranilate pathway based on a secured shikimate high-production strain (Lee et al., 2021). The present study performed additional engineering to produce anthranilate starting from the shikimate-overproducing strain. The further re-designed anthranilate-producing *E. coli* strain produced approximately 4 g/L of shikimate in 7-L fed-batch fermentation. These results suggest that the artificial cell factory design for the shikimate-overproducing strain would be valuable for constructing a microorganism-based strain for producing aromatic compounds in industrial quantities.

## Materials and methods

### Bacterial strains, media, and culture conditions

Table 1 lists all the bacterial strains used in this study. *E. coli* DH5α was used as the cloning host, and *E. coli* AB2834 was used as the metabolite production host. All *E. coli* strains were grown in Luria-Bertani (LB) medium at 30 or 37°C with the appropriate antibiotics. Small-scale cultivation and fed-batch fermentation for anthranilate production were conducted, as described previously (Lee et al., 2021). For small-scale cultivation in a 24-well culture plate, a single colony was inoculated in 1.3 ml LB medium at 30°C for 15 h. The culture broth was inoculated with 1% (v/v) in the same medium at 30°C for 15 h. The secondary culture broth was inoculated in 1.3 ml of *E. coli* production medium (EPM) at 30°C for 4 days. The miniature cultivation was performed using a humidity chamber set to 80% humidity and 200 rpm.

**Table 1:** Strains and plasmids used in this study.

Strain or plasmid	Characteristics	Sources or reference
*Escherichia coli* AB2834		
Inha215	AB2834△*tyrR*△*ptsG*△*pykA* △*lacI::Plac_aroB_aroD_Plac_aroG_aroF_Plac_ppsA_galP* △*aroL*△*aroK*△*shiA* / p*PoppA-aroE-tktA*	Lee et al. (2021)
Inha250	Inha215△*aroL::ParoL-aroL_ParoK-aroK*△*trpD*△*pheA&tyrA*	This study
Inha251	Inha215△*aroL::ParoL-aroL_ParoK-aroK*△*trpD*△*pabA*	This study
Inha252	Inha215△*aroL::ParoL-aroL_ParoK-aroK*△*trpD*△*ubiC*	This study
Inha253	Inha215△*aroL::ParoL-aroL_ParoK-aroK*△*trpD*△*entC*	This study
Inha254	Inha215△*aroL::ParoL-aroL_ParoK-aroK*△*trpD*△*pheA&tyrA* △*pabA*△*ubiC*△*entC*	This study
Inha255	Inha215△*aroL::ParoL-aroL_ParoK-aroK*△*trpD*△*pheA&tyrA* △*pabA*△*ubiC*△*entC*△*menF*	
Inha256	Inha254△*aroL::PJ23119_aroL&aroK*△*trpR* △*aroE_AB2834_::PoppA_aroE_K-12_*△*tktA::PJ23119_tktA*	This study
Inha257	Inha256△*pabB*△*menD*△*aroA::PoppA_aroA&aroC*	This study
Plasmid		
pCas	*repA101*(Ts) *kan Pcas-cas9 ParaB-Red lacI*q *Ptrc*-sgRNA-*pMB1*	Addgene (Jiang and Zhang, 2016)
pTargetF	*pMB1 aadA* sgRNA-*cadA*	Addgene (Jiang and Zhang, 2016)
pTarget-*PJ23119_aroL-aroK*	pTargetF containing sgRNA of *aroL*, constitutive J23119 promoter, coupled *aroL* & *aroK gene* and its homologous arms	This study
pTarget-*trpR*	pTargetF containing sgRNA of *trpR* and its homologous arms	This study
pTarget-*PoppA_aroE*	pTargetF containing sgRNA of *aroE*, *oppA* promoter, *aroE* gene, and its homologous arms	This study
pTarget-*PJ23119_tktA*	pTargetF containing sgRNA of *tktA*, constitutive *J23119* promoter, *tktA* gene, and its homologous arms	This study
pTarget-*trpD*	pTargetF containing sgRNA of *trpD* and its homologous arms	This study
pTarget-*pheA-tyrA*	pTargetF containing sgRNA of *pheA* and its homologous arms	This study
pTarget-*pabA*	pTargetF containing sgRNA of *pabA* and its homologous arms	This study
pTarget-*pabB*	pTargetF containing sgRNA of *pabB* and its homologous arms	This study
pTarget-*entC*	pTargetF containing sgRNA of *entC* and its homologous arms	This study
pTarget-*ubiC*	pTargetF containing sgRNA of *ubiC* and its homologous arms	This study
pTarget-*menD*	pTargetF containing sgRNA of *menD* and its homologous arms	This study
pTarget-*PoppA_aroA-aroC*	pTargetF containing sgRNA of *aroA*, *oppA* promoter, coupled *aroA & aroK* gene and its homologous arms	This study

### Construction of plasmids and strains

Table 1 lists the constructed plasmids, and Supplementary Table 1 presents all primer pairs used in this study. The CRISPR/Cas system was utilized for targeted gene editing. The two-plasmid system, in which the cas9 gene and a targeting N_20_ sequence directing it to the gene loci of interest, were separated in the pCas and pTargetF, respectively. N_20_ sequences followed by the PAM were designed by the web tool, CHOPCHOP.^1^ The homologous DNA fragments to the upstream and downstream regions of the target gene were amplified with primer sets. The fragment, including the N_20_ sequence and guide RNA scaffold, was also amplified. These fragments were cloned into *SpeI/HindIII*-digested pTargetF based on the In-Fusion Cloning method (TaKaRa, Japan). For gene overexpression, PJ23119 or PoppA promoter sequence and target gene amplified with the specific primers were cloned with homologous DNA fragments and N_20_ sequence into pTargetF simultaneously. Genome editing and plasmid curing were performed, as described previously (Jiang and Zhang, 2016). The transformants were verified by colony PCR and DNA sequencing. TransStart® FastPfu Fly DNA polymerase (Transgen Biotech., China) and SapphireAmp® Fast PCR Master Mix (TaKaRa, Japan) were used to amplify the target-specific fragments and colony PCR, respectively.

### Fed-batch fermentation

For fed-batch fermentation, the single colony was inoculated in 5 ml LBG (25 g/L LB broth and 20 g/L glucose) medium at 30°C, 200 rpm for 12 h. The primary culture broth was inoculated with 1% (v/v) in 20 ml same medium at 30°C for 6 h. The secondary culture broth was then inoculated with 1% (v/v) in a 7 L fermenter. The production culture was performed using the modified PB4-md5 medium. The modified PB4-MD5 medium included the following: 30 g/L glucose, 10 g/L glycerol, 37.125 g/L yeast extract, 5.25 g/L KH_2_PO_4_, 1 g/L MgSO_4_∙7H_2_O, 2 ml/L trace metals, and 200 μg/L thiamine hydrochloride. The feeding medium was composed of 600 g/L glucose, 100 g/L yeast extract, 20 g/L MgSO_4_∙7H_2_O, and 5 ml/L trace metal. Phosphate was not added to the feeding medium to regulate cell growth. The feeding medium was supplied using a peristaltic pump when glucose was depleted. The pH was adjusted with 10 N NaOH or 3 M HCL at 7.0, and the DO level was maintained above 40% by controlling the rpm, aeration, and feeding rates (Lee et al., 2021).

### Anthranilate and shikimate pathway metabolites analyses

The cultured broth was centrifuged at 15,000 RPM for 5 min. The supernatant was filtered using a 0.22 μm syringe filter (Rainbow PVDF membrane filter) and stored at −20°C. The concentrations of anthranilate and the shikimate pathway metabolites were determined by high-performance liquid chromatography (HPLC) using a Zorbax SB-Aq column (4.6 × 250 mm, Agilent, USA). The mobile phase was 0.1% trifluoroacetic acid (TFA) in 30% MeOH, and the flow rate was 0.5 ml/min. Anthranilate and shikimate pathway metabolites were detected at 330 and 214 nm, respectively.

## Results

### Redesign of anthranilate biosynthetic pathway in shikimate-overproducing *E. coli*

The shikimate over-producing strain (Inha215) obtained in a previous study (Lee et al., 2019) was an engineered *E. coli* cell factory strain designed to maximize the accumulation of shikimate by deleting six genes (*tyrR*, *ptsG*, *pykA*, *shiA*, *aroL*, and *aroK*) from the chromosome and by overexpressing eight genes (*aroB*, *aroD*, *aroF*, *aroG*, *galP*, *ppsA*, *aroE*, and *tktA*; Table 1). An attempt was made to re-activate the shikimate pathway by compensating for the shikimate kinase genes (*aroL* and *aroK*) involved in the conversion of shikimate to shikimate-3-phosphate. Using the CRISPR/Cas9 system, two genes, *aroL* and *aroK*, were complemented to be expressed under their own-promoter regulation. Unlike the shikimate over-producing Inha 215 strain, which could not synthesize the essential aromatic amino acids, this complemented strain was activated by shikimate kinases and grew well in minimal medium (Supplementary Figures 1A,B).

Anthranilate is a metabolite produced by converting chorismate by an anthranilate synthase (TrpEG) in the shikimate pathway (Figure 1), which is then converted to an anthranilate-phosphate precursor by anthranilate phosphoribosyltransferase, TrpD (Xie et al., 2003; Merino et al., 2008; Balderas-Hernández et al., 2009). Therefore, by deleting *trpD* in the presence of *aroL* and *aroK*, anthranilate was accumulated successfully without other metabolic processes (Supplementary Figures 1C,D). In addition, there are several enzymes involved in bypass routes for chorismate to be converted to other metabolites, including bifunctional chorismate mutase/prephenate dehydratase (encoded by *pheA* and *tyrA*), anthranilate synthase (encoded by *trpEG*), ρ-aminobenzoate synthase (encoded by *pabAB*), isochorismate synthase (encoded by *menF* and *entC*), chorismate lyase (encoded by *ubiC*) (Figure 1, Dosselaere and Vanderleyden, 2001; Sprenger, 2007; Noda et al., 2016). The genes involved in the bypass routes mentioned above were removed sequentially to maximize the accumulation of chorismite as much as possible. Based on the shikimate over-producing Inha215 strain, Inha250 with the *pheA* and *tyrA* genes removed, Inha251 with the *pabA* and *tyrA* gene removed, Inha252 with the *entC* gene removed, and Inha254 strain with the *pheA*, *tyrA*, *pabA*, *entC*, and *ubiC* gene removed were generated sequentially (Supplementary Figures 1C,D). Finally, approximately 18.7 mg/L of anthranilate was produced into the Inha254 strain (Supplementary Figure 1E). The Inha255 strain, which also removed *menF* encoding isochorismate synthase in *E. coli*, was also developed, but unlike the Inha254 strain, anthranilate production was not carried out (Supplementary Figure 1E).

### Redesign of the shikimate pathway genes to enhance anthranilate production

**Figure 2:**
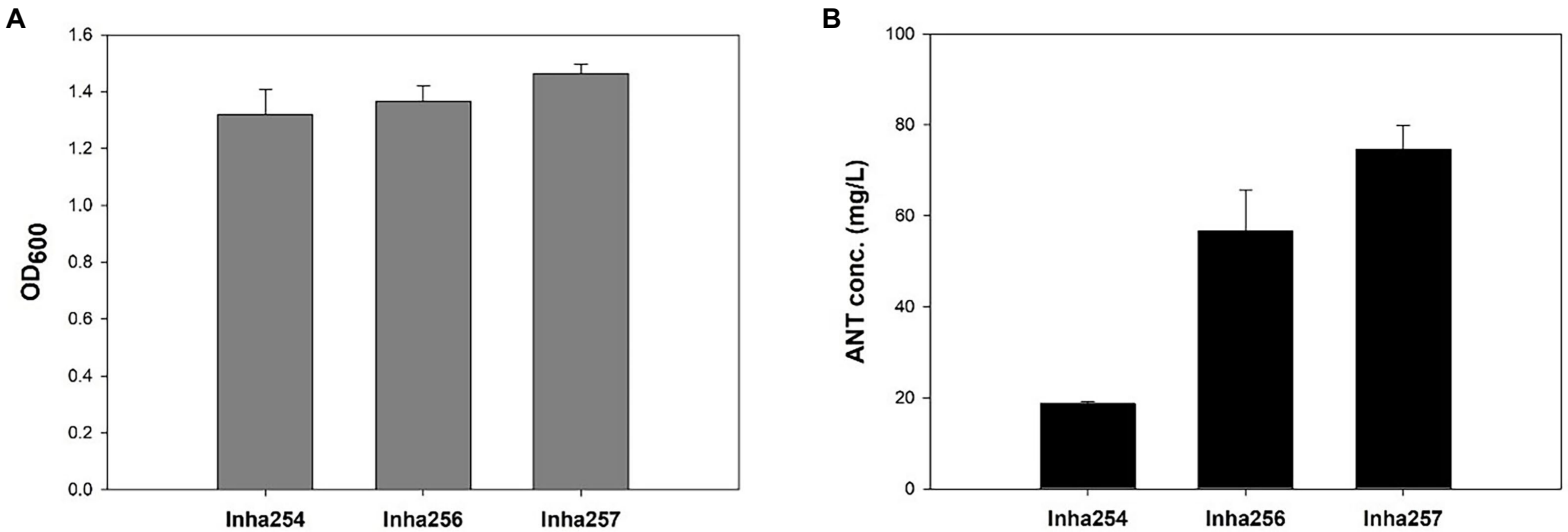
Comparison of cell growth and anthranilate production yields in recombinant *E. coli* strains (Inha254, Inha256, and Inha257) at 72 h miniature cultivation. **(A)** Cell growth as measured by OD600. **(B)** Quantitative analysis of anthranilate production yield.

Five key shikimate pathway genes, such as *trpR*, *aroE*, *tktA*, *aroL*, and *aroK*, were engineered further to maximize anthranilate production using the Inha254 strain constructed above. First, a well-known shikimate pathway transcriptional regulator gene, *trpR*, was deleted from the Inha254 chromosome, and the *aroE* was integrated into the Inha254 chromosome under the control of strong *oppA* promoter (Supplementary Figures 2, 3; Gu et al., 2016). Second, to increase the erythrose-4-phosphate (E4P) pool, one of the essential precursors of the shikimate pathway, the *tktA* involved in the pentose phosphate pathway was over-expressed under the control of the strong promoter J23119 (Supplementary Figure 4; Shen et al., 2012; Lee et al., 2021). Third, the *aroL* and *arolK* genes were coupled translationally and expressed under the control of the strong promoter J23119 (Supplementary Figure 5). The strain called Inha256, which was optimized for these five genes (*trpR*, *arooE*, *tktA*, and *aroL&aroK*) produced 56.8 mg/L of anthranilate, which is approximately three times higher production yield than that of Inha254 (Figure 2).

Moreover, to minimize the chorismite bypass pathways, *menF* encoding 2-succinyl-5-enolpyruvyl-6-hydroxy-3-cyclohexene-1-carbosylate synthase and *pabB* encoding ρ-aminobenzoate synthase subunit involved in the chorismite metabolism to either isochorismate and p-aminobenzoate, respectively (Supplementary Figure 6). In addition, two genes, *aroA* and *aroC*, were translationally coupled and overexpressed under the control of strong *oppA* promoter (Supplementary Figure 7). Approximately 74.6 mg/L of anthranilate was produced in the strain described above (called Inha 257; Figure 2).

### Fed-batch fermentation for anthranilate production

Although the anthranilate production yield of the Inha257 strain increased 1.3 fold compared to the Inha256 strain, the cell growth of Inha257 also increased 1.1 fold, suggesting that the redesign of the four genes (*menD*, *fabB*, *aroA*, and *aroC*) attempted in the Inha257 had a positive effect on cell growth rather than anthranilate production optimization (Figure 2). Occasionally, the inha257 strain was inconsistent in both cell growth and anthranilate productivity compared to the Inha256 strain (data not shown), so that the subsequent fed-batch experiment was conducted using the Inha256 strain.

**Figure 3:**
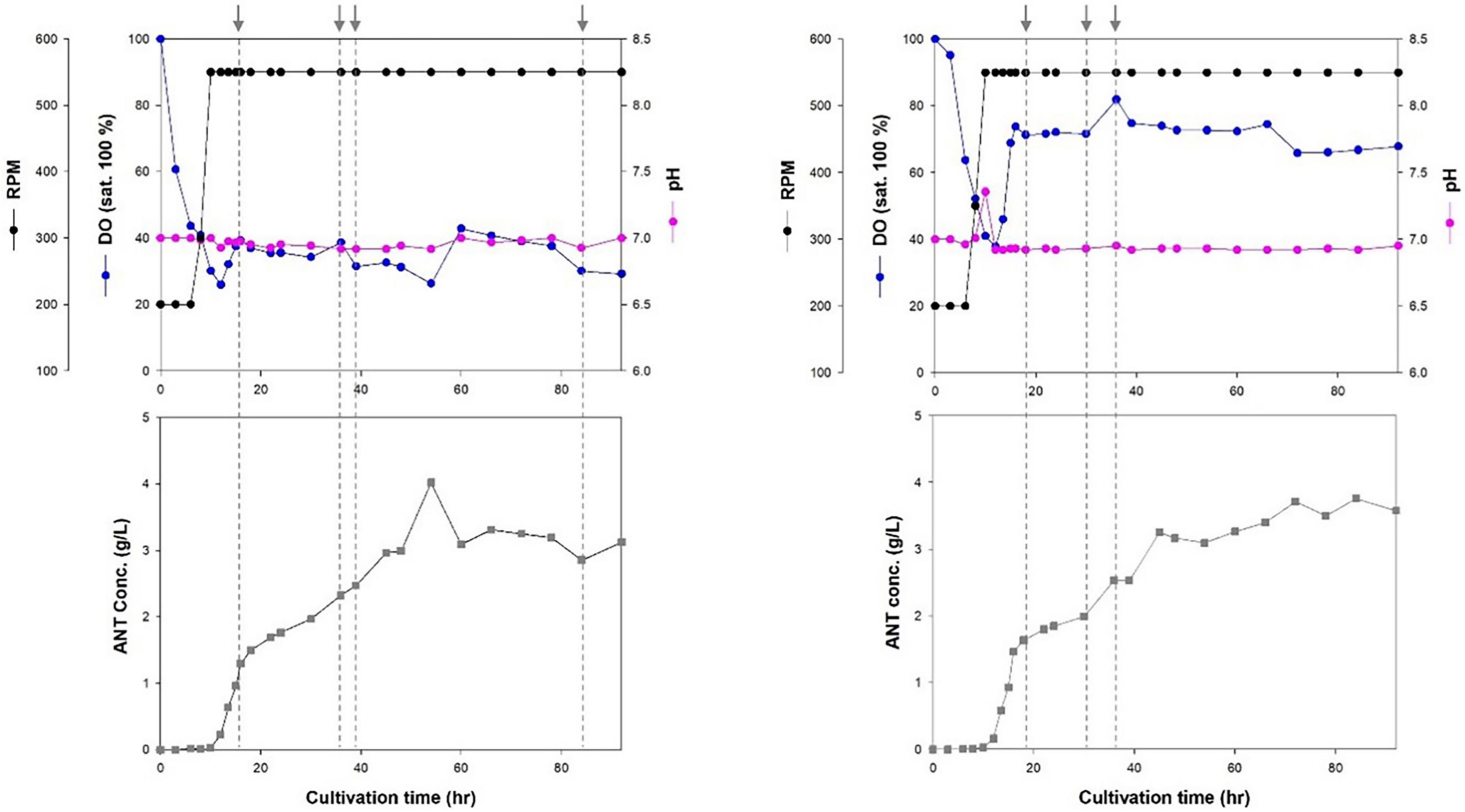
Fed-batch fermentation for anthranilate production of Inha256 in a 7 l bioreactor. Time course of RPM, DO concentration, pH and anthranilate production yield were indicated, respectively. The feeding (indicated as an arrow) was started with 0.1323 ml/min at 16 h and changed with 0.1701 ml/min, 0.1512 ml/min and 0.0567 ml/min at 36, 39, and 84 h, respectively **(Left)**. The feeding was started with 0.1323 ml/min at 16 h and changed with 0.0945 and 0.1323 ml/min at 30 and 36 h, respectively **(Right)**.

Cell growth increased rapidly after 8 h of incubation. The stationary phase was reached after 18 h in a 7-L fed-batch fermentation of Inha256 strain (Figure 3). Subsequently, cell growth slightly increased after 27 h of culture with a lower feeding rate, but showed constant cell growth until 72 h when the culture was terminated. The production yield of anthranilate in the Inha256 strain gradually increased after 10 h of culture and reached the maximum after 60 h of culture, producing approximately 4 g/L in the recombinant *E. coli* (Figure 3). Interesting, however, 18 g/L of anthranilate was once observed depending on the culture and feeding conditions (Supplementary Figure 8).

## Discussion

This study attempted to construct an anthranilate high-production recombinant *E. coli* strain through genetic and metabolic engineering. Based on the shikimate over-producing strain obtained in previous studies, the shikimate pathway was reinforced, and the metabolic pathway was redesigned to accumulate the main precursor, chorismate, in the biosynthesis pathway of anthranilate. In strengthening the shikimate pathway, the efficient expression of *aroE* encoding a shikimate dehydrogenase, is believed to be an important step. Unlike previous studies that expressed *aroE* in a high-copy vector (Lee et al., 2021), this study applied the CRISPR/Cas9 system to generate a plasmid-free expression system by designing *aroE* to be expressed stably on *E. coli* chromosome under the control of a powerful OppA promoter.

In addition, all chorismate bypass routes, except the anthranilate biosynthesis pathway, were blocked to accumulate chorismate, a key precursor of an anthranilate. Interestingly, when *menF* encoding isochorismate synthase was removed, virtually no anthranilate and shikimate production was observed, suggesting that MenF interacted with other shikimate pathway-related enzymes. The additional removal of *menD* encoding a 2-succinyl-5-enolpyruvyl-6-hydroxy-3-carbosylate synthase and *pabB* encoding a p-aminobenzoate synthase subunit were pursued to maximize the accumulation of menaquinone and polyacids from the chorismate. Surprisingly, there were no significant changes in the chorismate accumulation (Supplementary Figure 9). Therefore, it was confirmed that the removal of the aforementioned five genes, such as *pheA*, *tyrA*, *pabA*, *ubiC*, *entC*, and *trpR*, was the best option to maximize the accumulation of anthranilate. Although isoenzyme, AroL, is expected to act as the main shikimate kinase by showing a substrate affinity approximately 100 times higher than AroK. Moreover, the activity of AroL is reported to be greatly affected by the type and concentration of divalent cation (Huang et al., 1975; DeFeyter and Pittard, 1986). Therefore, additional anthranilate over-production could be achieved through AroL enzyme engineering and kinetics optimization.

In conclusion, the rationally designed anthranilate-overproducing *E. coli* strain grown in an optimized medium produced approximately 4 g/L of anthranilate in 7-L fed-batch fermentation. Overall, rational cell factory design and culture process optimization for microbial-based anthranilate production will play a vital role in complementing traditional chemical-based anthranilate production processes.

## Data availability statement

The raw data supporting the conclusions of this article will be made available by the authors, without undue reservation.

## Author contributions

SL and E-SK designed the research. H-JK, S-YS, H-SP, J-YK, and S-SC performed the experiments, as well as data collection and analysis. H-JK and J-YK performed genetic engineering. S-YS and H-SP performed medium optimization and fermentation. H-JK, J-YK, S-SC, SL, and E-SK wrote and edited the manuscript. All authors contributed to the article and approved the submitted version.

## Funding

This study was carried out with the support of “Cooperative Research Program for Agriculture Science and Technology Development (project no. PJ01563901)” Rural Development Administration.

## Conflict of interest

S-YS and SL are employed by STR Biotech Co., Ltd.

The remaining authors declare that the research was conducted in the absence of any commercial or financial relationships that could be construed as a potential conflict of interest.

## Publisher’s note

All claims expressed in this article are solely those of the authors and do not necessarily represent those of their affiliated organizations, or those of the publisher, the editors and the reviewers. Any product that may be evaluated in this article, or claim that may be made by its manufacturer, is not guaranteed or endorsed by the publisher.

## Footnotes

^1^https://chopchop.cbu.uib.no
