# Supplementary material for: Engineered Escherichia coli cell factory for anthranilate over-production
Source: Front Microbiol. 2023 Mar 15;14:1081221. doi: 10.3389/fmicb.2023.1081221 (PMC10050376; doi:10.3389/fmicb.2023.1081221)
Supplement: Supplementary file 1 [file Data_Sheet_1.PDF]

Engineered *Escherichia coli* Cell Factory for Anthranilate Over-production

Hye-Jin Kim[1], Seung-Yeul Seo[2], Heung-Soon Park[1], Ji-Young Ko[1], Si-Sun Choi[1], Sang Joung Lee[2] and Eung-Soo Kim[1]*

[1]Department of Biological Sciences and Bioengineering, Inha University, Incheon 22212, Korea

[2]STR Biotech Co., Ltd., Bioplaza 4-3, 56, Soyanggang-ro, Chuncheon-si, Gangwon-do, 24232, Korea

*Corresponding author: Eung-Soo Kim

Tel: 82-32-860-8318

Fax: 82-32-872-4046

E-mail: eungsoo@inha.ac.kr

Keywords: Anthranilate, metabolic pathway engineering, *Escherichia coli*, genome editing, fed-batch fermentation

Supplemental Figure 1. Development of the anthranilate producing strain based on shikimate overproducing strain (Inha215) (A) Scheme of shikimate kinase genes (*aroL* and *aroK*) complementation, (B) Cultivation of strains complemented shikimate kinase genes, (C) Scheme of the *trpD* gene and chorismate metabolic genes deletion, (D) DNA sequencing results to verify removal of target gene in Inha250~255, (E) Anthranilate production yield in Inha254 and Inha255 strains.

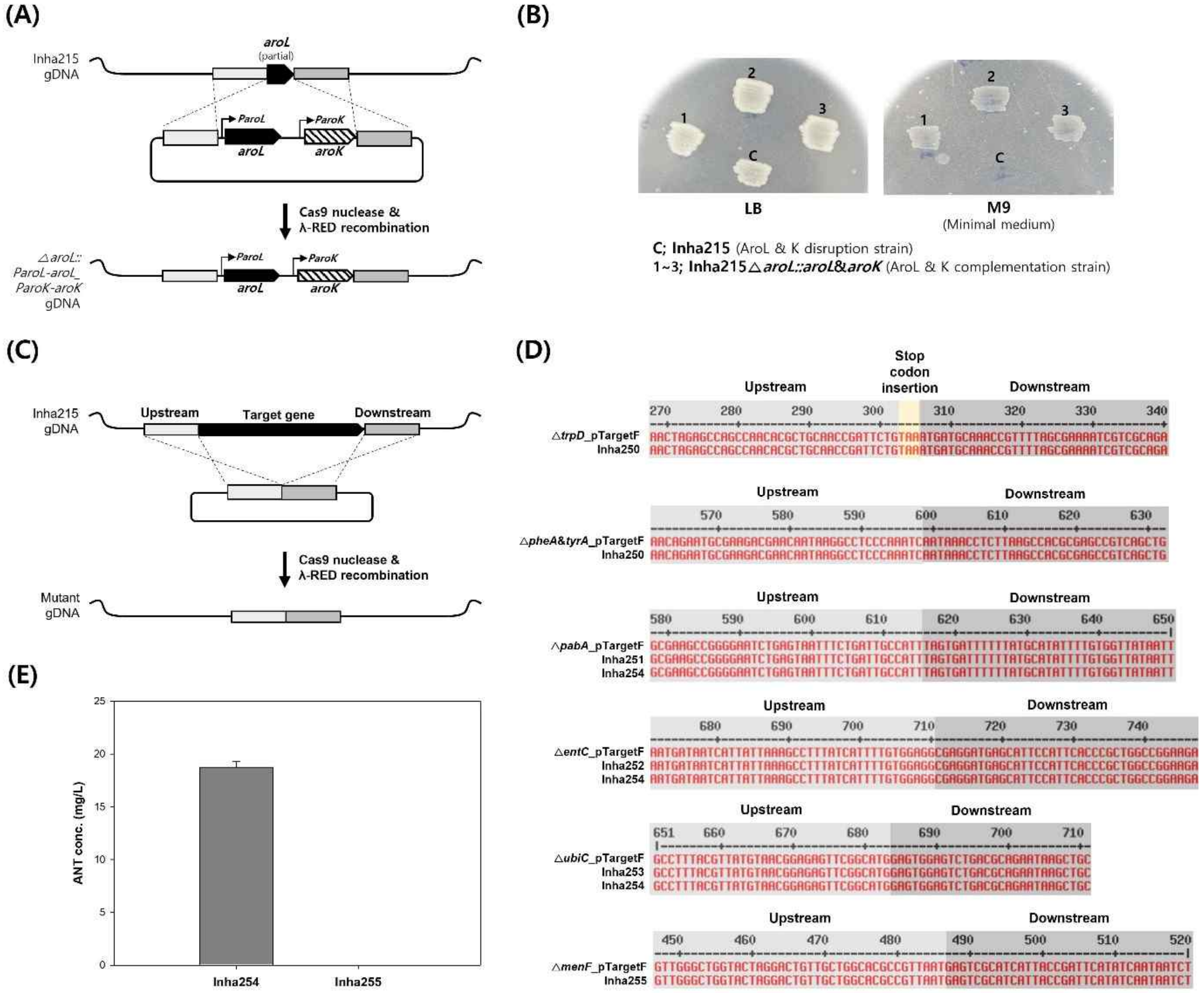

Supplemental Figure 2. Development of anthranilate producing strain (Inha256) through disruption of DNA-binding transcriptional repressor (encoded by *trpR* gene). (A) Scheme for deletion of *trpR* gene, (B) DNA sequencing results to verify *menF* gene deletion in Inha256.

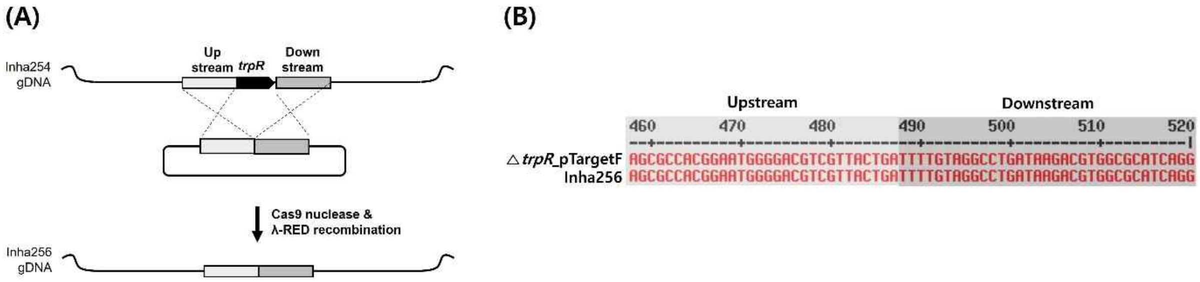

Supplemental Figure 3. Substitution of AB2834-derived shikimate dehydrogenase (encoded by *aroE* gene) with *E. coli* K-12-derived shikimate dehydrogenase under the control of the *oppA* promoter. (A) Scheme for substitution of *aroE* gene, (B) DNA sequencing results to verify inactive *aroE* gene substitution with wild-type *aroE* gene in Inha256.

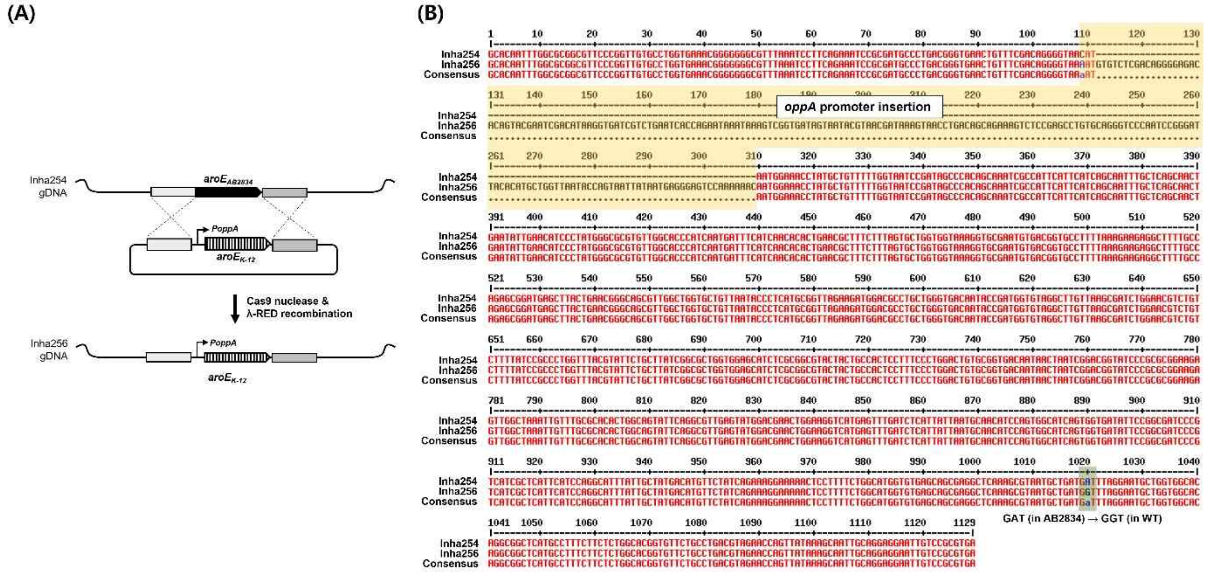

Supplemental Figure 4. Additional insertion of the J23119 promoter and RBS for efficient expression of transketolase (encoded by *tktA* gene). (A) Scheme for insertion of *J23119* promoter and RBS in front of *tktA* gene, (B) DNA sequencing results to verify *J23119* promoter and RBS insertion in Inha256.

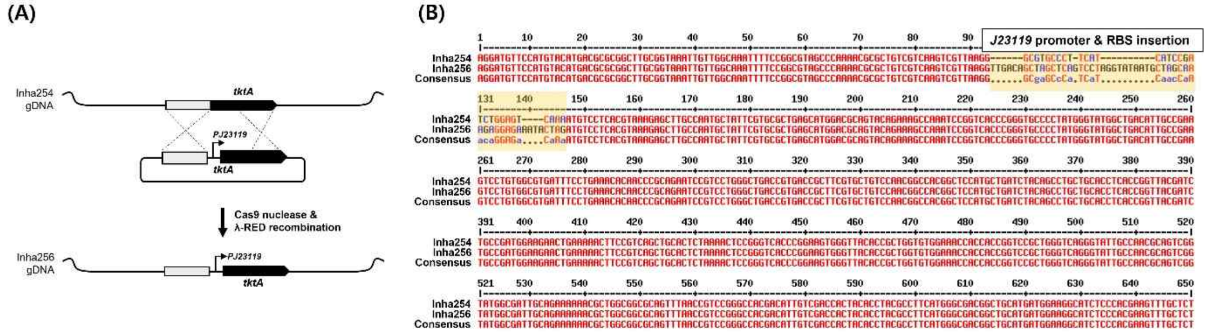

Supplemental Figure 5. Optimization for efficient expression of shikimate kinases (encoded by *aroL and aroK* genes). (A) Scheme for insertion of *J23119* promoter and coupled shikimate kinase genes, (B) DNA sequencing results to verify *J23119* promoter and coupled shikimate kinase genes insertion in Inha256.

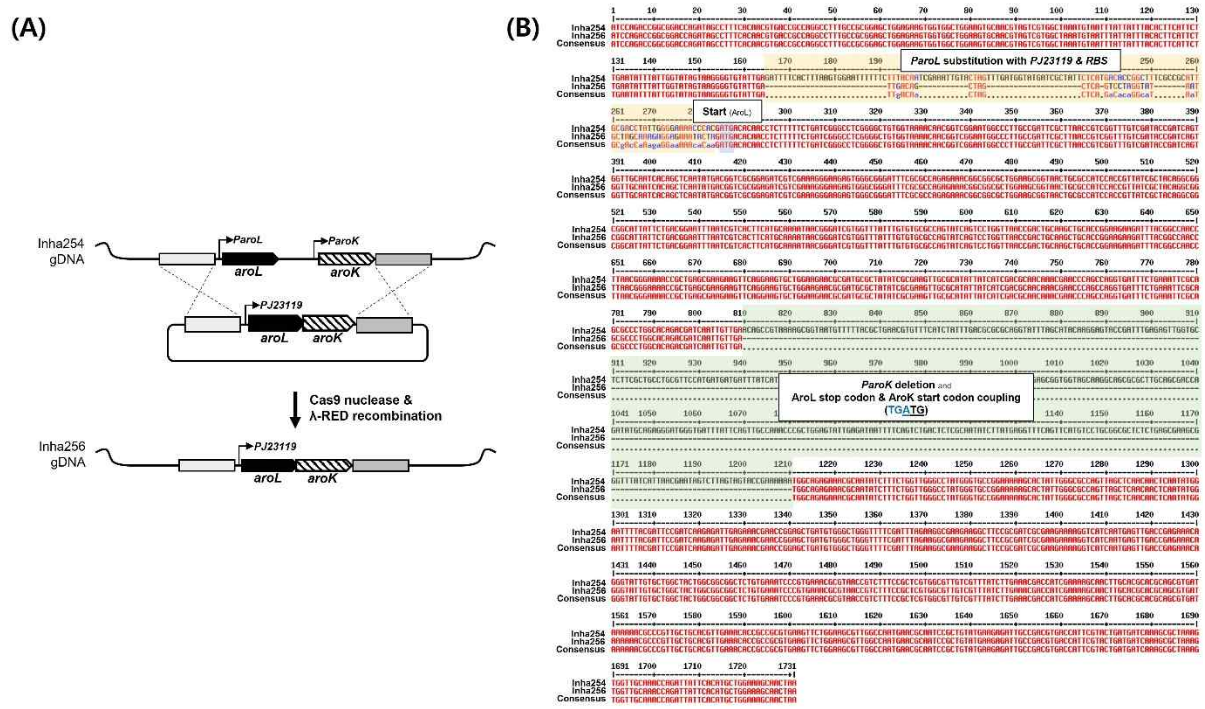

Supplemental Figure 6. Additional disruption of chorismate metabolic pathway through deletion of 2-succinyl-5-enolpyruvyl-6-hydroxy-3-cyclohexene-1-carboxylate synthase (encoded by *menD* gene) and ρ-aminobenzoate synthase subunit (encoded by *pabB* gene). (A) Scheme for deletion of *menD* and *pabB* genes, (B) DNA sequencing results to verify targeted gene deletion in Inha257.

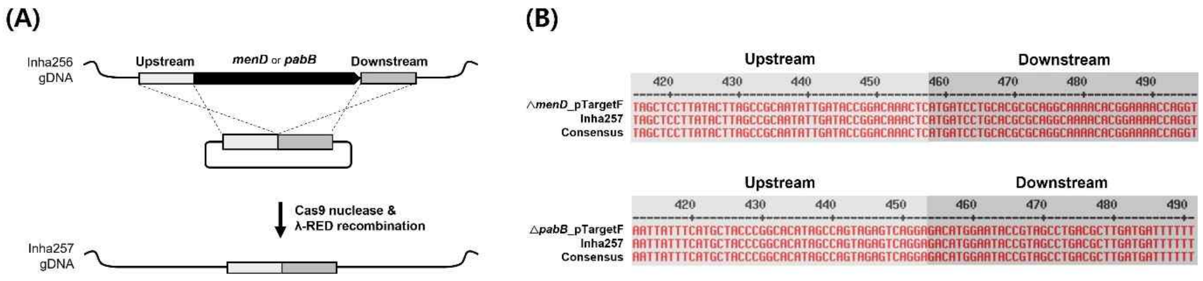

Supplemental Figure 7. Optimization for efficient expression of 3-phosphoshikimate-1-carboxyvinyltransferase (encoded by *aroA* gene) and chorismate synthase (encoded by *aroC* gene). (A) Scheme for insertion of *oppA* promoter and coupled *aroA* & *aroC* genes, (B) DNA sequencing results to verify *oppA* promoter and coupled *aroA* & *aroC* genes insertion in Inha257.

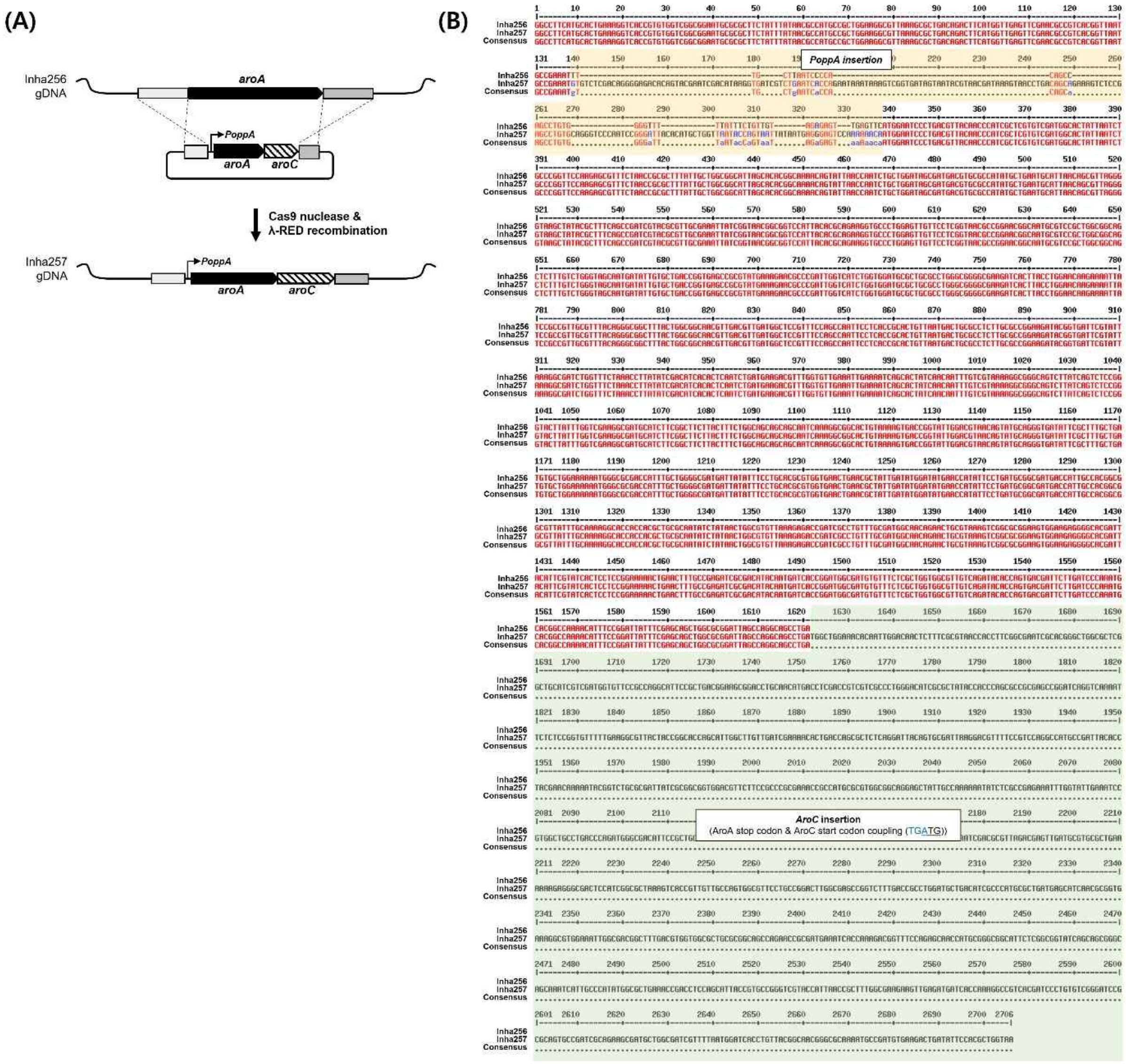

Supplemental Figure 8. Fed-batch fermentation for anthranilate production in a 7 L bioreactor. Time course of RPM, DO concentration, pH, DCW, and anthranilate production yield were indicated, respectively. The feeding was started with 0.1701 ml/min at 15 hr and changed with 0.1323 and 0.1701 ml/min at 27 and 42 hr, respectively.

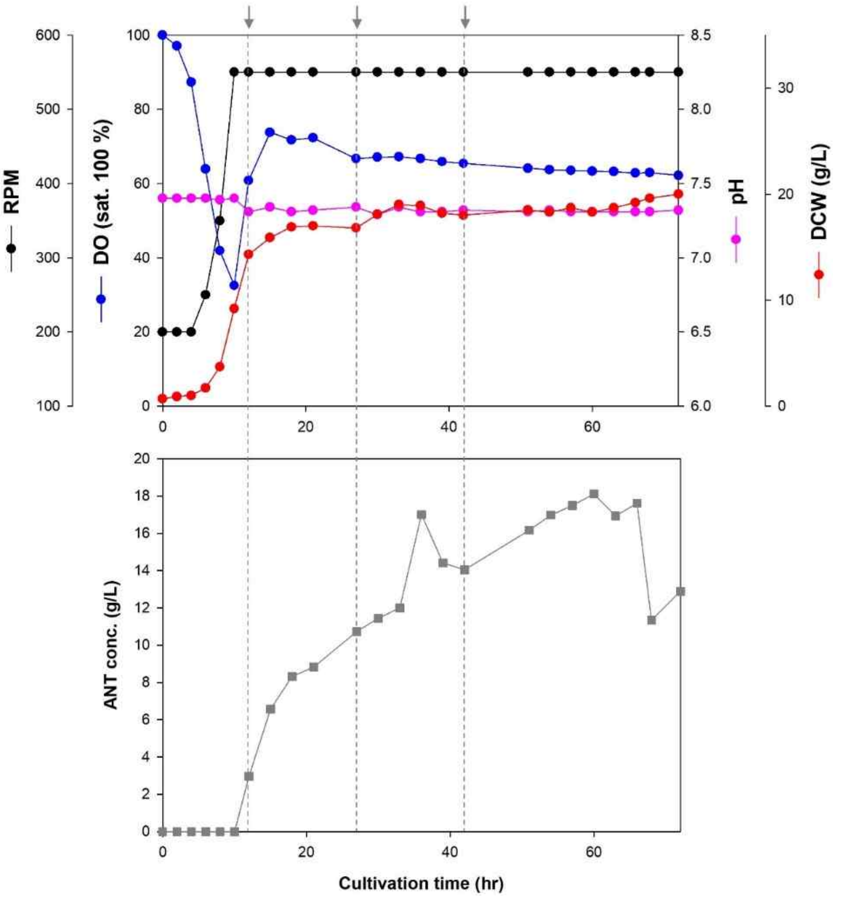

Supplemental Figure 9. Accumulation of chorismate in Inha256 and Inha257 strains.

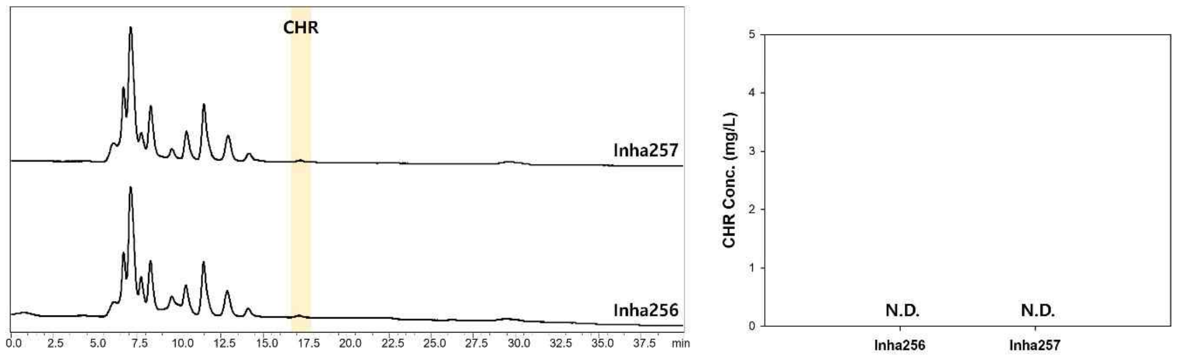

Supplemental Table 1. Primer pairs for the construction of plasmids and target gene amplification used in this study.

Purpose		Forward(F)<br>Reverse(R)
aroL and *aroK* complementation	sgRNA scaffold	5'-agtcctaggtataatactagtgcttaaccgtgaacccagccgttttagagctagaaatagcaag-3'
		5'-aagcttctgcaggtcgact-3'
	Upstream & *aroL*	5'-gacctgcagaagcttgacgcgtgtcccaatgtaat-3'
		5'-cgcttttacggctgttcaacaattgatcgtctgtgcc-3'
	aroK	5'-acagccgtaaaagcggtaatg-3'
		5'-ttagttgctttccagcatgtg-3'
	Downstream	5'-ctggaaagcaactaacgtgaaataaggaagaacgatg-3'
		5'-cagggtaatagatctaagcttgttcatccaccttactttt-3'
	Complement CK	5'-ccaacaaaggatgactttatgacaatt-3'
trpD deletion	sgRNA scaffold	5'-gtcctaggtataatagaattctgtcaggccaaagtcttctggttttagagctagaaatagcaa-3'
		5'-aagcttctgcaggtcgact-3'
	Upstream	5'-agggtaatagatctaaagcttctccacggtaaagcctcc-3'
		5'-aacggtttgcatcatttacagaatcggttgcagcg-3'
	Downstream	5'-atgatgcaaaccgttttagc-3'
		5'-gagtcgacctgcagaaagctttgacgatggggaggaaatta-3'
	Deletion CK	5'-gcgattgtcgaagcttacgg-3'
pheA and *tyrA* deletion	sgRNA scaffold	5'-agtcctaggtataatactagttccgcacctgataacccgaggttttagagctagaaatagc-3'
		5'-aagcttctgcaggtcgact-3'
	Upstream	5'-agagtcgacctgcagaagcttaacatgtcgcagaccgtct-3'
		5'-gatttgggaggccttattgttc-3'
	Downstream	5'-aaggcctcccaaatcaataaacctcttaagccacgcg-3'
		5'-cagggtaatagatctaagcttgcaacagcaattaacgctatgc-3'
	Deletion CK	5'-ttgacatcaggaacggtatgc-3'
pabA deletion	sgRNA scaffold	5'-agtcctaggtataatactagtcagcgaatggtagcgtgtcagttttagagctagaaatagc-3'
		5'-aagcttctgcaggtcgact-3'
	Upstream	5'-agagtcgacctgcagaagcttatttgcgaactatccatcgcc-3'
		5'-aatggcaatcagaaattactcagattccccggctt-3'
	Downstream	5'-tttctgattgccatttagtgatttttta-3'
		5'-cagggtaatagatctaagctttaaacagcgagcgaccatga-3'
	Deletion CK	5'-cttgatatcatgcgtaaccgg-3'
pabB deletion	sgRNA scaffold	5'-taggtataatactagtgaccactgatgacccgctacgttttagagctagaaatagc-3'
		5'-aagcttctgcaggtcgact-3'
	Upstream	5'-gacctgcagaagcttatggagacaggtgaacgttc-3'
		5'-acggtattccatgtctcctgactctactggctatg-3'
	Downstream	5'-gacatggaataccgtagcctgacg-3'
		5'-taatagatctaagcttgccatacccgatgtgaatca-3'
	Deletion CK	5'-tgaccaggaaggctggaac-3'
entC deletion	sgRNA scaffold	5'-agtcctaggtataatactagttgaggaagtacagcagaccagttttagagctagaaatagc-3'
		5'-aagcttctgcaggtcgact-3'
	Upstream	5'-agagtcgacctgcagaagcttcacggctgtcagtaatctga-3'
		5'-gaatgctcatcctcgcctccacaaaatgataaaggctt-3'
	Downstream	5'-cgaggatgagcattccattc-3'
		5'-cagggtaatagatctaagcttgaatggaggaatgttctgtgac-3'
	Deletion CK	5'-tcatccgcgacgcggttatt-3'
menF deletion	sgRNA scaffold	5'-agtcctaggtataatactagtgcaatcacttactacggcgcgttttagagctagaaatagc-3'
		5'-aagcttctgcaggtcgact-3'
		5'-agagtcgacctgcagaagcttactggcctgataagcctgtttac-3'

	Upstream	5'-ttaacggcgtgccagcaac-3'
	Downstream	5'-ctggcacgccgttaatgagtcgcatcattaccgattc-3'
		5'-cagggtaatagatctaagcttcggcaatgaaatactgtgcg-3'
	Deletion CK	5'-gatgaactggtggcgtatgc-3'
menD deletion	sgRNA scaffold	5'-taggtataatactagtccgtttgctgaaccgctgtagttttagagctagaaatagc-3'
		5'-aagcttctgcaggtcgact-3'
	Upstream	5'-gacctgcagaagcttgtactgcgtctgcgtaaagt-3'
		5'-cgcgtgcaggatcatgagtttgtccggtatcaatattg-3'
	Downstream	5'-atgatcctgcacgcgcag-3'
		5'-taatagatctaagcttgcgttgatcgtcattgagtg-3'
	Deletion CK	5'-catgctggtggtggaagata-3'
ubiC deletion	sgRNA scaffold	5'-agtcctaggtataatactagtgcgtgacgatgatccgcgaagttttagagctagaaatagc-3'
		5'-aagcttctgcaggtcgact-3'
	Upstream	5'-agagtcgacctgcagaagcttcagcgcttacatgatatgttgaa-3'
		5'-catgccgaactctccgttac-3'
	Downstream	5'-gagtggagtctgacgcagaa-3'
		5'-cagggtaatagatctaagcttagccattggaatcgaccagc-3'
	Deletion CK	5'-gagtggattcgtagtgatgatg-3'
trpR deletion	sgRNA scaffold	5'-gtcctaggtataatactagtgatggcagaacagcgtcaccgttttagagctagaaatagc-3'
		5'-aagcttctgcaggtcgact-3'
	Upstream	5'-gagtcgacctgcagaagctttacaccagcggtaaggagat-3'
		5'-tcagtaacgacgtccccatt-3'
	Downstream	5'-ggacgtcgttactgattttgtaggcctgataagacgt-3'
		5'-agggtaatagatctaagcttacgaaatcgggtagccaatg-3'
	Deletion CK	5'-gatcatctggaagagcgattc-3'
aroE substitution	sgRNA scaffold	5'-agtcctaggtataatactagtcaggggtaacataatgaatcgttttagagctagaaatagc-3'
		5'-aagcttctgcaggtcgact-3'
	Upstream	5'-agagtcgacctgcagaagcttagtgatgcgactgttggagt-3'
		5'-ctgtcgagacacattttacccctgtcgaaacag-3'
	oppA promoter	5'-aatgtgtctcgacaggggag-3'
		5'-agcataggtttccattgtttttggactccctca-3'
	aroE	5'-atggaaacctatgctgtttttgg-3'
		5'-cagggtaatagatctaagcttggcgcaatagtagaagatgatgt-3'
	Substitution CK	5'-acctgcaaagagacgctatc-3'
tktA promoter insertion	sgRNA scaffold	5'-gtcctaggtataatactagtaggacattttgactccagatgttttagagctagaaatagc-3'
		5'-aagcttctgcaggtcgact-3'
	Upstream	5'-gagtcgacctgcagaagcttcctagcgcattggcaatagt
		5'-tgagctagctgtcaaccttaacgacttgacgacag-3'
	PJ23119_tktA	5'-ttgacagctagctcagtcctaggtataatgctagcaaagaggagaaatactagatgtcctcacgtaaagagctt-3'
		5'-agggtaatagatctaagcttaacttcgtgggagatgcctt-3'
	Insertion CK	5'-ggaaggtttgtcagtcactg-3'
aroL and *aroK* coupling	sgRNA scaffold	5'-gtcctaggtataatactagtgggtgtattgacgtgaaatagttttagagctagaaatagcaag-3'
		5'-aagcttctgcaggtcgact-3'
	Upstream	5'-gacctgcagaagcttgacgcgtgtcccaatgtaat-3'
		5'-ggactgagctagctgtcaatcaatacaccccttactatacc-3'
	PJ23119_aroL	5'-ttgacagctagctcagtcctaggtataatgctagcaaagaggagaaatactagatgacacaacctctttttctgatc-3'
		5'-gcgtttctctgccatcaacaattgatcgtctgtgcc-3'
	aroK	5'-atggcagagaaacgcaatatctt-3'
		5'-ttagttgctttccagcatgtg-3'
	Downstream	5'-ctggaaagcaactaacgtgaaataaggaagaacgatg-3'
		5'-cagggtaatagatctaagcttgttcatccaccttactttt-3'
aroA and *aroC*		5'-agtcctaggtataatactagtaggcagcctgaatgaacaacgttttagagctagaaatagcaag-3'

coupling	sgRNA scaffold	5’-aagcttctgcaggtcgact-3’
	Upstream	5’-agagtcgacctgcagaagctttcgtcatcgttcgtgaagat-3’
		5’-cccctgtcgagacacatttcggcattaaccgtgacg-3’
	oppA promoter	5’-aatgtgtctcgacaggggag-3’
		5’-tgtttttggactccctcattataatt-3’
	aroA	5’-ggagtccaaaaaacaatggaatccctgacgttacaac-3’
		5’-caggctgcctggctaatc-3’
	aroC	5’-tagccaggcagcctgatggctggaaacacaattggac-3’
		5’-ttaccagcgtggaatatcagtc-3’
	Downstream	5’-attccacgctggtaacgggcaataaatagccaaatctt-3’
		5’-cagggtaatagatctaagcttacacgaacacagccgttcg-3’
	Insertion CK	5’-attgacgtcagccgttatgg-3’
